# Genomic Markers of Heavy Alcohol Consumption Outperform Self-Report and Other Common Predictors of Mortality in Two Middle-Aged Samples of Black Americans

**DOI:** 10.3390/genes17070799

**Published:** 2026-07-13

**Authors:** Steven R. H. Beach, Man-Kit Lei, Mei Ling Ong, Rachael D. Weaver, Sierra E. Carter, Robert A. Philibert

**Affiliations:** 1 Department of Psychology, The University of Georgia, Athens, GA 30602, USA; csierra@uga.edu; 2 Center for Family Research, The University of Georgia, Athens, GA 30602, USA; karlo@uga.edu (M.-K.L.); tmlong@uga.edu (M.L.O.); 3 Department of Sociology, The University of Georgia, Athens, GA 30602, USA; rachael.weaver@uga.edu; 4 College of Medicine, University of Iowa, Iowa City, IA 52242, USA; robert-philibert@uiowa.edu

**Keywords:** heavy alcohol consumption, Chronic Illness, mortality, BMI

## Abstract

Background/Objectives: Heavy alcohol consumption (HAC) may contribute to elevated mortality risk in middle age, but its contribution to mortality in middle age among Black Americans is poorly characterized, suggesting the potential for genomic variables to improve prediction. We examined whether a DNA methylation-based measure of HAC (HAC/ATS) increased prediction of mortality beyond Chronic Illnesses and other controls and compared its predictive abilities to that of self-reported HAC. Methods. Sample: Two longitudinal samples were examined to test hypotheses. First, we examined the Family and Community Health Study (FACHS) sample, comprising 360 individuals who were 95.8% female, with a mean age of 47.12 at the time of their blood draw, and followed for 17 years. Second, we examined the Promoting Strong African American Families (ProSAAF) sample comprising 336 individuals who were 54.8% female, with a mean age 44.64 at the time of their initial blood draw, and followed for 5 or more years. To overcome potential biases arising from small sample sizes we used Firth’s penalized likelihood logistic regression analyses to predict mortality in both samples. Results: In both samples, the DNA-methylation-based indicator of HAC (HAC/ATS) significantly contributed to the prediction of mortality beyond the significant contribution of age, Number of Chronic Illnesses, and other control variables. This was in contrast to the non-significant contribution of self-reported HAC. In both samples, high blood pressure was also a significant predictor of mortality, albeit not as powerful a predictor as DNA-methylation-based indicator of HAC. Conclusions: A DNA-methylation-based indicator of HAC (HAC/ATS) made a substantial contribution to mortality risk in two longitudinal samples of middle-aged African Americans. Significant contributions of Chronic Illness suggested the value of future exploration of genomic indicators of HAC in combination with chronic conditions.

## 1. Introduction

Chronic or heavy alcohol consumption (HAC) is a known risk factor for many diseases [1], and heavy drinking is relatively common in the United States. Approximately 80% of all adults in the US report consuming alcohol at some point in their lives, with 65% reporting use in the past year. Corresponding to increasing prevalence of alcohol consumption [2], there has been an increasing trend in alcohol-attributable mortality and alcohol-induced mortality, suggesting substantially increased impact of alcohol on mortality over the past 20 years [3]. In addition, although there has been some debate about the potential health benefits of low levels of alcohol consumption for health in the general population [4], there has been consistent agreement about the risks associated with HAC. However, the extent to which HAC contributes to mortality in mid-life among Black American men and women is poorly characterized, and its potential as a targetable health behavior to extend life and health span among middle-aged Black Americans may have been underestimated due to reliance on self-report indices of alcohol consumption. In addition, Black Americans do not always demonstrate the same patterns of alcohol consumption or association with problematic outcomes as do other groups. Although Black Americans are more likely to abstain from alcohol and generally report lower overall alcohol use, there are subgroup- and life-course-specific patterns of persistent or later-peaking heavy episodic drinking, alongside disproportionately high alcohol-related consequences among Black drinkers, resulting in the potential for greater health and mortality impact than would be anticipated on the basis of overall consumption alone [5].

Black Americans also suffer from greater prevalence and earlier onset of Chronic Illness than other ethnic groups and suffer greater comorbidity among Chronic Illnesses [6,7]. Although Chronic Illnesses may begin to emerge in young adulthood, the most prominent race-related differences in health and mortality emerge in midlife (35–65), with particularly disturbing trends observed for mortality among middle-aged Black women since 1990 [8]. These trends suggest that better understanding predictors of middle-age mortality among Black Americans is particularly important from a public health perspective. Unfortunately, although HAC has been identified as one potential predictor of mortality [9], the full extent of its role may be obscured by a tendency toward underreporting HAC on self-report indices, a problem that may be more pronounced among those with higher levels of HAC [10], i.e., the people of greatest interest for prediction of health and mortality outcomes. Self-report of alcohol consumption, particularly at higher levels, may be underestimated for several reasons, including stigma, recall problems, difficulty in accurately estimating consumption, and deliberate underestimation, and it does not appear that self-report can be easily corrected by statistical means alone [11]. This suggests the value of characterizing alcohol’s impact on mortality using non-self-report indices of HAC [12,13].

Despite recognition of the role of HAC in health and mortality outcomes, the magnitude of those contributions to midlife mortality among Black Americans may be underappreciated. We test the hypothesis that HAC is a central, modifiable exposure relevant to prediction of midlife mortality whose impact may be better appreciated when non-self-report indices are utilized. This perspective has direct implications for prevention science, perhaps particularly in healthcare settings serving adults with chronic disease where targeted alcohol screening and intervention may yield disproportionate benefits for mortality reduction. If a reliable measure of HAC predicts mortality in midlife, HAC can be more readily targeted as a preventable exposure, providing a point of intervention for potential population-level improvements in health outcomes.

We focus on HAC, measured via non-self-report, DNA methylation (DNAm)-based methods, as a predictor of mortality risk over relatively short prospective intervals among two samples of middle-aged African Americans. Using two samples allows us to examine effects across a broader range of midlife and to examine effects for both men and women. We also contrast the impact of HAC to BMI, Self-reported smoking, and diagnosed Chronic Illness, highlighting the potential additive value of non-self-report indices of HAC relative to other widely used predictive measures. In supplemental analyses we show the relative impact of self-reported HAC compared to non-self-reported indices of HAC (HAC/ATS), underscoring the likely incremental value of non-self-report measures of HAC.

### Specific Hypotheses

(H1) In a sample of primarily female, middle-aged African American adults (the FACHS sample), using the Illumina InfiniumMethylationEpic Beadchip array (a.k.a. the EPIC array), methylation values associated with the Alcohol T-score (ATS) were used to characterize heavy alcohol consumption (HAC) (i.e., cg04987734, cg04583842, cg02583484, cg09935388). We expected that greater HAC would predict increased mortality even after considering associations with age, sex, BMI, number of chronic conditions, and smoking.

(H2) We expected that follow-up analyses with the FACHS sample examining individual chronic conditions would also show that HAC (HAC/ATS) was a stronger predictor than presence vs absence of any of the individual chronic conditions often associated with mortality included in the chronic condition index.

(H3) In a sample of both male and female, younger midlife African American adults (the ProSAAF sample), using drop digital assessment of methylation values associated with the ATS to characterize HAC, we expected to confirm that HAC (HAC/ATS) predicted mortality even after considering associations with age, sex, BMI, number of chronic conditions, and smoking.

(H4) We expected that follow-up analyses with the ProSAAF sample examining individual chronic conditions would show that HAC (HAC/ATS) was a stronger predictor than any of the specific chronic conditions and would remain a significant predictor of mortality even when all predictors were included in the model.

In supplemental analyses we also directly tested potential differences in prediction of mortality using self-reported HAC vs. non-self-report indices.

## 2. Materials and Methods

All procedures were approved by the Institutional Review Board at the University of Georgia. For study 1, which used FACHS primary caregivers (PCs), participants were primarily female. IRB approval was under the title “FACHS weathering—Protocol study number 00004856.” For study 2, using relatively equal numbers of male and female adult participants in the ProSAAF sample, all procedures were also approved by the University of Georgia, IRB approval number 2012104 112. Analyses were not preregistered. Materials are available by request from the corresponding author. Analytic code is provided in Appendix B. Due to potential identifiability of the data, they are not publicly available. AI was not used to generate text, data, or graphics, or to assist in study design, data collection, analysis, or interpretation.

### 2.1. Study Participants

The Family and Community Health Study (FACHS). We examined our hypotheses using data collected from the N = 361 PCs for the FACHS project who provided blood and were successfully assayed for DNAm at Wave 5 in a longitudinal investigation of health and development among Black American families residing in Iowa and Georgia or adjacent communities. One additional participant was lost due to having missing BMI at Wave 5, yielding a final analytic sample of N = 360. HAC was assessed in 2008 when blood-based biomarker data collection was introduced. Accordingly, we used the sample of 360 participants at Wave 5, mean age 47.12 (SD = 8.119), 95.8% female, for whom we could determine a blood-based index of HAC at Wave 5 and who did not have missing data on any other Wave 5 covariates being examined, and for whom we could determine mortality status. All FACHS primary caregivers at Wave 1 were followed to determine impact on mortality through 6-18-2025, allowing for a 17-year follow-up period. More detail on the sample, including both earlier and later waves of assessment can be found in Lei [14], and in the Appendix C. A total of 43 participants were deceased as of 2025. The mortality status of all participants included in the analytic sample was determined.

The Promoting Strong African American Families (ProSAAF) project. All hypotheses for sample two were examined using data collected for the ProSAAF sample from 2019 to 2020 at Wave 5 (six years after initial participation in the project) when blood-based biomarker data collection was introduced. This yielded a sample of N = 336 individuals, mean age 44.64 (SD = 8.265)**,** who could be followed to determine the contribution of HAC (HAC/ATS) to mortality status five or more years later determined between 3 and 10 and 2025 and 4-1-2025. More detail on the sample, including both earlier and later waves of assessment can be found at [15] and in the Appendix C. Of the participants, 152 participants were male (45.2%) and 184 participants were female 54.8%, with 14 participants deceased as of 2025. Nine participants were not included in the analyses because mortality status could not be determined.

### 2.2. Procedures

All participants provided informed consent, and the University institutional review board approved all study procedures and protocols. For FACHS, all Wave 5 interviews were conducted in participants’ homes by a pair of interviewers using computer-assisted interviews that lasted about two hours. For ProSAAF, all Wave 5 interviews were conducted in the participants’ homes using a computerized survey. Surveys required approximately 1 h.

### 2.3. Study Measures

Covariates. To address potential alternative explanations, analyses controlled for Wave 5 chronological age, self-reported sex, number of chronic conditions often associated with increased mortality (cancer, liver disease, heart disease, diabetes, high blood pressure, and kidney disease), BMI, and smoking in all analyses. Control variables were entered in the Firth penalized regression models in the same way they would be in a standard logistic regression.

Chronic Conditions. Participants were asked about whether they had ever received a diagnosis for a list of 27 chronic conditions. Specifically, they were asked: “Has a doctor ever told you that you were suffering from…”. Those most likely to contribute to mortality were the focus of the comparative analyses. Accordingly, number of chronic conditions was a count of cancer, liver disease, heart disease, diabetes, high blood pressure, and kidney disease. In follow-up analyses, chronic conditions were examined as simultaneous individual predictors in each sample.

BMI. Respondents were asked to report their height and weight, and this information was used to calculate a Body Mass Index (BMI) score. BMI score was calculated according to the following formula provided by the Center for Disease Control [16]: weight (lbs.)/height in inches^2^ × 0.703 [17].

Self-reported smoking. As the leading overall cause of preventable death [18] cigarette smoking was also included as a predictor of mortality. Participants were asked, in FACHS, “On average, how many cigarettes do you usually smoke per day?”, and in ProSAAF, “In the past month, how often did you smoke cigarettes?”.

### 2.4. Biodata Collection

To collect blood samples, a certified phlebotomist visited the home and collected 4 tubes of blood for FACHS participants and 6 tubes of blood for ProSAAF participants (30 mL total in each case). Blood draws were non-fasting. Blood samples were delivered to the Psychiatric Genetics Lab at the University of Iowa. DNA was prepared via standard procedures and stored at −20 °C until used.

Epigenome-wide methylation assessments were conducted using Illumina InfiniumMethylationEpic Beadchip array (a.k.a. EPIC) by the University of Minnesota Genome Center according to the manufacturer’s (Illumina, San Diego, CA, USA) instructions. Samples were randomized on arrays to minimize batch effects. The resulting data were DASEN normalized using the MethyLumi [19], WateRmelon [20] and IlluminaHumanMethylationEPICanno.ilm10b2.hg19 (Illumina) R packages [21,22]. CpG values were background-corrected using the “noob” method. Finally, data were subject to standard sample- and probe-level quality control measures.

In the FACHS PC sample, the Alcohol T-score (ATS) for each individual was assessed using Illumina EPIC array data. Accordingly, the ATS W5 score for the FACHS sample was calculated using the method described by Miller et al. [23] in which the key CpG sites linked to chronic HAC, that are available in the EPIC array, were combined as ATSW5 = ((cg04987734 + cg04583842 − cg02583484 − cg09935388)/4).

In ProSAAF, HAC/ATS was assessed at Wave 5 using methylation-sensitive digital PCR (MSdPCR) designed specifically to assess the ATS, providing more precise measurement. Values were an unweighted sum of the four z-scores of MSdPCR assays obtained at same four loci previously shown to reflect variation in heavy alcohol use (cg02583484, cg04987734, cg09935388 and cg04583842) [23]. The ATS analyses for ProSAAF were performed by staff blinded to participants’ other study variables to ensure analytical independence.

Mortality Assessment. Mortality data for participants in the FACHS and ProSAAF samples were searched using the systematic death index in the “Advanced Person Search” in the Accurint database. The FACHS sample was assessed between 10 June 2025 and 4 December 2025, while the ProSAAF sample was assessed between 11 March 2025, and 31 March 2025. We employed several verification processes, including relatives report, full names, dates of birth, sex, and residential addresses, to ensure high-confidence identification. Mortality was treated as a binary variable. Individuals confirmed as deceased during the assessment were coded as 1, individuals alive were coded as 0, and those with insufficient identifying information or database limitations were coded as 99 and excluded from the analysis as missing data.

### 2.5. Statistical Analyses

Correlations among predictors, mortality, and covariates, along with means and SDs for continuous variables, were computed first for descriptive purposes. Percent and point-biserial correlations were computed for dichotomous variables (e.g., sex, presence vs. absence of specific chronic conditions). We used Firth penalized logistic regression to estimate the association between HAC/ATS and mortality occurring between Wave 5 (2018–2020) and the 2025 mortality assessments, while adjusting for potential confounding variables. This approach addresses small-sample bias and standard-error inflation problems commonly encountered in standard Cox models [24]. Standard maximum likelihood estimation (MLE) displays a bias toward zero and often leads to non-convergence [25,26]. In contrast, Firth’s method produces more stable and accurate estimates [27], overcomes convergence issues [28], and improves the prediction of relatively infrequent events [29]. The Firth penalized logistic regression produces a pseudo R-square statistic to indicate fit. A higher pseudo R-square means the model with predictors fits the mortality outcome better than a model with no predictors, but the value is not directly interpretable as explained variance.

## 3. Results

### 3.1. Descriptive Findings for FACHS and ProSAAF Samples

General associations and descriptive statistics for the FACHS and ProSAAF samples are shown in Table 1, which provides correlations, means, and Standard Deviation for the primary study variables. As can be seen in Table 1, for the FACHS PC sample, mean age was 47.12 (SD = 8.119). BMI was elevated on average, with mean BMI = 35.02 (8.73), and much of the sample reported one or more Chronic Illnesses (38.89% reporting 1, 18.33% reporting more than 1). The most commonly reported chronic conditions of those examined were high blood pressure (49.7%), diabetes (15%), and heart disease (10.6%)]. Somewhat less common were cancer (4.4%), kidney disease 3.6%, and liver disease (1.7%). Age was robustly correlated with Number of Chronic Illnesses (r = 0.355, *p* = 0.001), ATS (r = 0.174, *p* = 0.001), and mortality (r = 0.470, *p* = 0.001), but not with BMI, smoking, or self-reported binge drinking. However, BMI was significantly correlated with mortality (r = −0.130, *p* = 0.013).

As can also be seen in Table 1, there were similar patterns of association in the ProSAAF sample. Mean age was 44.64 (SD = 8.265). BMI was elevated on average with mean BMI = 34.69 (9.072), and much of the sample reported one or more Chronic Illnesses (33.04% reporting 1, 17.56% reporting more than 1). The most commonly reported chronic conditions of those examined were high blood pressure (45.24%), diabetes (16.67%), and heart disease (6.55%), with cancer, liver disease, and kidney disease all less than 4%. As was the case in the FACHS sample, age was robustly correlated with Number of Chronic Illnesses (r = 0.276, *p* = 0.001), ATS (r = 0.139, *p =* 0.011), and mortality (r = 0.145, *p* = 0.008), but not with BMI, smoking, or Self-reported heavy alcohol consumption. Number of Chronic Illnesses was significantly correlated with mortality (r = 0.136, *p* = 0.012).

Appendix A Table A1 and Table A2 provide correlations between predictors and the individual chronic conditions, and also provide correlations with Self-reported heavy alcohol consumption for both FACHS (Appendix A Table A1) and ProSAAF (Appendix A Table A2). Appendix A Table A3 also provides the correlations with treatment condition in the ProSAAF sample, showing that treatment condition was not significantly associated with other study variables.

### 3.2. H1

HAC/ATS will predict mortality in a primarily female, middle-aged African American sample over a nine-year period even after controlling for age and other risk factors.

Firth analyses for the FACHS sample. Firth penalized regression analyses predicting mortality for the FACHS sample are reported in Table 2. As can be seen in Table 2, in all models, age was a significant predictor of mortality (all *p*’s < 0.001). The DNAm index of HAC (HAC/ATS) was also a significant predictor of mortality when it was entered in model 1 and model 3 (*p*’s < 0.001). HAC/ATS was a stronger predictor of mortality than either BMI (*p* < 0.05 in model 2) or number of diagnosed Chronic Illnesses. Surprisingly, Self-reported smoking was not significantly associated with increased mortality risk in this middle-aged sample.

### 3.3. H2

HAC/ATS will outperform individual Chronic Illnesses and conditions in predicting mortality when all are considered separately.

As can be seen in Table 3, when we examined all chronic conditions separately in the FACHS sample rather than examining count of comorbid conditions, HAC/ATS, continued to be a significant predictor of mortality (Model 1 and Model 3). However, of the specific chronic conditions, only high blood pressure accounted for significant unique additional variance in mortality (*p* = 0.031).

In Table A4, we examined the impact of including self-reported HAC (SRHAC) on mortality in the FACHS sample, and found that it accounted for no additional significant variance in mortality beyond the effect of control variables, and did not add to prediction of mortality using HAC/ATS. This is consistent with the performnace of self-reported HAC at the level of simple correlations shown in Table 1, where it was not significantly associated with mortality. Conversely, HAC/ATS robustly predicted mortality beyond the effects of self-reported HAC (*p* < 0.01).

### 3.4. H3

The ATS will continue to predict mortality in the ProSAAF sample, a slightly younger sample of middle-aged Black Americans more evenly divided between men and women.

As can be seen in Table 4, Model 1, BMI was a significant predictor of mortality (*p* < 0.027) in the ProSAAF sample before number of chronic conditions was added to the model. However, after the addition of number of chronic conditions in models 2 and 3, the effect of BMI dropped to non-significance; whereas, the effect of Number of Chronic Illnesses was significant. The ATS was robustly associated with mortality both with and without number of chronic conditions in the model (Models 1 and 3), *p* < 0.001.

### 3.5. H4

The ATS will outperform Chronic Illnesses in predicting mortality over a six-year period, even when Chronic Illnesses are considered separately.

As can be seen in Table 5, BMI was a significant predictor of mortality (*p* < 0.049) in the baseline model but became a non-significant predictor when specific chronic conditions were added to the baseline model (Model 3). The ATS was a robust predictor of mortality (*p* < 0.001), both with and without specific chronic conditions in the model. Of the chronic conditions, having received a diagnosis of cancer, high blood pressure, and kidney disease all contributed significant unique variance in model 3.

In supplemental analyses (Appendix A Table A5) we examined the predictive utility of self-reported HAC in the ProSAAF sample and found that it did not account for significant variance in mortality in the baseline model and also did not account for significant variance in models including either ATS or number of chronic disorders or both. However, both ATS and number of chronic disorders continued to account for significant unique variance (*p* < 0.001 and *p* < 0.05, respectively). This pattern was not changed using specific conditions in the model and controlling self-reported HAC for the ProSAAF sample (Appendix A Table A6) or when treatment condition, i.e., assignment to a relationship enhancement program or not six years earlier, was included as an additional predictor in the ProSAAF sample (Appendix A Table A7). In each case HAC/ATS was a significant predictor, whereas the self-report HAC measure (SRHAC) was not a significant predictor of mortality.

## 4. Discussion

Our findings indicate that heavy alcohol consumption (HAC), as indicated by ATS scores, predicts mortality risk among middle-aged Black Americans, and that the prediction of mortality risk is improved by using DNAm-based markers rather than self-reported HAC. Across two middle-aged Black American samples followed over different time frames, one slightly older and almost entirely female and the other having more equal numbers of male and female participants, we found that, whereas self-reported HAC was a non-significant predictor of mortality, a non-self-report index developed to capture methylation-based change linked to HAC (HAC/ATS), was consistently a substantial and significant predictor of mortality. In addition, we found that HAC/ATS outperformed smoking, Chronic Illness, and BMI as predictors of excess mortality. As expected, age was a consistent predictor of mortality in both Black middle-aged samples and so was an appropriate control in all analyses. Second, in supplemental analyses examining the six chronic conditions comprising our count of Chronic Illnesses individually, high blood pressure was a significant predictor of mortality net of other chronic conditions, age, and HAC/ATS in both middle-aged samples; whereas, cancer and kidney disease were additional predictors of mortality in the slightly younger sample with relatively equal numbers of males and females. These results suggest the potential for joint effects and highlight the importance of considering HAC/ATS alongside the presence of Chronic Illnesses, particularly those related to cardiovascular and renal function, when assessing midlife mortality risk.

At the population level, alcohol is a major contributor to global mortality and disability, accounting for millions of deaths annually across cardiovascular disease, cancer, liver disease, injuries, and infections [30,31]. Notably, global burden analyses consistently show that alcohol ranks among the leading risk factors for death and disability in younger adults, in contrast to older ages where chronic disease burden dominates irrespective of behavioral exposures [30]. However, the current findings suggest that alcohol may be particularly relevant for understanding elevated mortality risk in the 35–65-year age range among Black Americans, even when accounting for the burden of the number of chronic conditions.

Interpretation of the alcohol–mortality association has also been shaped by longstanding debates surrounding the supposed benefits of low-level consumption and its potential cardioprotective effects. However, recent high-quality meta-analytic work that explicitly addresses key sources of bias, such as misclassification of former drinkers as abstainers and residual confounding, has challenged the robustness of purported protective effects of moderate drinking for all-cause mortality [9]. When such biases are considered, the overall relationship between alcohol and mortality appears more consistently harmful across a wide range of consumption levels, but particularly for heavy alcohol consumption. In parallel, and consistent with the current findings, large registry-based and cohort studies demonstrate that alcohol use disorder (AUD) is associated with markedly elevated mortality risk across adulthood, with particularly pronounced hazard ratios observed in early and midlife [32].

At the same time, a growing body of evidence also suggests that alcohol may exert its most harmful effects in the presence of pre-existing Chronic Illness, potentially accelerating disease progression through multiple mechanisms, including systemic inflammation, metabolic dysregulation, direct organ toxicity, and carcinogenesis [31]. In addition, alcohol may destabilize chronic disease management by interacting with medications, impairing adherence, worsening sleep and mental health, and increasing the likelihood of acute events such as arrhythmias, hypertensive crises, and injuries. As a result, those with cardiovascular disease, hypertension, and cancer who were already at elevated mortality risk may suffer disproportionately from HAC [33,34,35]. However, HAC is particularly likely to be underreported among those with one or more diagnosed Chronic Illnesses, leading to difficulty in identification and intervention when self-report is used to index HAC in this context.

Some limitations to the current investigation should be noted. First, the relatively small sample size for each of the samples examined precluded meaningful analyses of interaction effects with contextual stressors and/or chronic conditions and other biological predictors. This underscores the need for replication with larger samples, allowing examination of the role of chronic stressors and inflammatory processes, two notable sources of health disparities, in contextualizing the current findings. Further, the current investigation focuses on the use of HAC/ATS to predict mortality two Black middle-age samples, accordingly further work showing the limits of generalizability to other population groups will be important. In addition, we focus on only one non-self-report index of HAC. Replication using other non-self-report indices of HAC is an important focus of future research. Likewise, the current findings do not explicate biological mechanisms that might be better characterized in samples with metabolomic or proteomic data sets to compliment patterns observed with methylation-based biomarkers. It should also be noted that the small sample sizes precluded good estimates of the magnitude of the incremental prediction afforded by HAC/ATS and this could be better determined in future studies with larger samples. Likewise, because of the relatively large portion of the sample that reported diagnosed Chronic Illness, it is likely that many were taking medications and the potential interaction of medications with HAC/ATS should also be further examined in larger samples. Finally, the current findings suggest, but do not demonstrate, that alcohol may be an actionable point of intervention. Direct examination of culturally sensitive interventions designed to address and reduce HAC are an important next step for future research.

## 5. Conclusions

These limitations notwithstanding, the current findings suggest that DNAm-based measures of HAC, particularly HAC/ATS, have advantages relative to self-reported HAC in identifying those most in need of, and most likely to benefit from, HAC reduction interventions. Accordingly, one potential implication of the current findings is that HAC should be assessed using DNAm-based methods in the context of general and specialty practices among those concerned that self-report may be providing an underestimate of use and limiting intervention options. Likewise, researchers examining the impact of HAC in the context of medically vulnerable populations may want to consider using DNAm markers as complementary assessment tools that can be used to help identify underreported exposure to HAC in research settings. For populations in which alcohol stigma is likely to be high, such as Black Americans or those in the Southeastern United States more generally, underreporting among those at greatest risk for harmful effects of HAC should be considered likely. Future work with larger, better-powered, and more diverse samples should examine potential interactions between DNAm HAC and specific chronic conditions and evaluate how underreporting on self-report measure may affect these associations. By highlighting the predictive power of non-self-report alcohol measures in an under-researched population, our results advance understanding of modifiable risk factors in midlife and point toward strategies to mitigate mortality disparities.

## Figures and Tables

**Table 1:** Descriptive statistics and correlations for study variables including demographic, control, predictor and outcome variables, with N = 360 for FACHS (below diagonal) and N = 336 for ProSAAF (above diagonal).

	Age	Sex	BMI	HAC/ATS	SR Smoke	SR HAC	N Illnesses	Mortality	Mean	SD
Age	—	0.211 **	−0.091 ^†^	0.139 *	−0.083	−0.081	0.276 **	0.145 **	44.644	8.265
Sex	0.004	—	−0.201 **	0.214 **	0.198 **	0.135 *	0.109 *	0.050	0.452	0.498
BMI	−0.067	0.004	—	−0.174 **	−0.191 **	−0.075	0.115 *	0.074	34.690	9.072
HAC/ATS	0.174 **	−0.013	−0.035	—	0.442 **	0.209 **	0.110 *	0.238 **	2.195	3.527
SRsmoke	−0.012	0.089 ^†^	−0.078	0.268 **	—	0.342 **	−0.009	−0.030	0.679	1.235
SRHAC	−0.030	0.093 ^†^	−0.082	−0.005	0.120 *	—	−0.032	−0.040	0.443	0.827
NIllnesses	0.355 **	0.005	0.039	0.107 *	−0.037	−0.003	—	0.136 **	0.226	0.419
Mortality	0.470 **	−0.034	−0.130 *	0.260 **	0.038	−0.053	0.236 **	—	0.042	0.200
										
Mean	47.125	0.042	35.018	−0.020	2.431	0.067	0.256	0.119		
SD	8.119	0.200	8.733	0.047	5.705	0.300	0.437	0.325		

† *p* < 0.10, * *p* < 0.05, ** *p* < 0.01. Note: correlation for ProSAAF display above the diagonal; correlation for FACHS display below the diagonal; means and SDs for ProSAAF display in the rightmost columns; means and SDs for FACHS display in the bottom rows; BMI = Body Mass Index; HAC/ATS = Alcohol T-score; SRsmoke = Self-reported smoking; SRHAC = Self-reported heavy alcohol consumption; NIllnesses = Number of Chronic Illnesses; SD = Standard Deviation. The Chronic Illnesses variable was calculated as the aggregate sum of six dichotomous indicators (1 = Yes, 0 = No): high blood pressure, cancer, liver disease, heart disease, diabetes, and kidney disease. ATS was calculated differently for FACHS and ProSAAF, placing them on different scales.

**Table 2:** Firth regression showing that HAC (ATS score) is a significant predictor of mortality, accounting for significant variance beyond age, sex, BMI, Self-reported smoking (SRsmoke), and Number of Chronic Illnesses (NIllness) in the FACHS sample (N = 360).

	Baseline	Model 1	Model 2	Model 3
	*β* (SE)	*β* (SE)	*β* (SE)	*β* (SE)
Age	1.162 **	1.085 **	1.023 **	0.929 **
	(0.178)	(0.184)	(0.186)	(0.191)
				
Sex	−0.125	0.253	−0.155	0.248
	(0.925)	(0.902)	(0.933)	(0.904)
				
Body Mass Index	−0.412	−0.363	−0.461 *	−0.415
	(0.216)	(0.215)	(0.225)	(0.223)
				
SRsmoke	0.196	−0.029	0.206	−0.012
	(0.159)	(0.191)	(0.154)	(0.184)
				
HAC/ATS		0.726 **		0.750 **
		(0.220)		(0.216)
				
NIllnesses			0.875	1.035 *
			(0.488)	(0.505)
				
Constant	−2.455 **	−2.593 **	−3.012 **	−3.269 **
	(0.226)	(0.246)	(0.422)	(0.455)
Pseudo-R^2^	3.455	3.593	4.012	4.269

* *p* < 0.05, ** *p* < 0.01. Note: SRsmoke = Self-reported smoking; HAC/ATS = Alcohol T-score; NIllnesses = Number of Chronic Illnesses.

**Table 3:** Firth regression showing that HAC (ATS score) is a significant predictor of mortality, accounting for significant variance beyond control variables and beyond the presence of six specific Chronic Illnesses in the FACHS sample: cancer, liver disease, heart disease, diabetes, high blood pressure, or kidney disease, with only blood pressure contributing additional unique variance (N = 360).

	Baseline	Model 1	Model 2	Model 3
	*β* (SE)	*β* (SE)	*β* (SE)	*β* (SE)
Age	1.162 ***	1.085 **	0.903 ***	0.855 ***
	(0.178)	(0.184)	(0.201)	(0.210)
				
Sex	−0.125	0.253	−0.016	0.350
	(0.925)	(0.902)	(0.927)	(0.905)
				
Body Mass Index	−0.412	−0.363	−0.482 *	−0.409
	(0.216)	(0.215)	(0.237)	(0.231)
				
SRsmoke	0.196	−0.029	0.188	−0.019
	(0.159)	(0.191)	(0.159)	(0.191)
				
HAC/ATS		0.726 **		0.660 **
		(0.220)		(0.222)
				
Cancer			1.421 *	1.234
			(0.613)	(0.644)
				
Liver			−0.771	−0.129
			(1.086)	(1.090)
				
Heart Disease			0.367	0.027
			(0.555)	(0.586)
				
Diabetes			0.336	0.337
			(0.478)	(0.500)
				
High Blood Pressure			0.912 *	0.979 *
			(0.449)	(0.454)
				
Kidney			−0.064	−0.243
			(0.802)	(0.878)
				
Constant	−2.455 ***	−2.593 ***	−3.140 ***	−3.236 ***
	(0.226)	(0.246)	(0.391)	(0.398)
Pseudo-R-squared	3.455	3.593	4.140	4.236

* *p* < 0.05, ** *p* < 0.01, *** *p* < 0.001. Note: SRsmoke = Self-reported smoking; HAC/ATS = Alcohol T-score.

**Table 4:** Firth regression showing that HAC (ATS score) is a significant predictor of mortality, accounting for significant variance beyond age, sex, BMI, Self-reported smoking (SRsmoke), and Number of Chronic Illnesses (NIllness) in the ProSAAF sample (N = 336).

	Baseline	Model 1	Model 2	Model 3
	*β* (SE)	*β* (SE)	*β* (SE)	*β* (SE)
Age	0.595 **	0.440	0.373	0.301
	(0.227)	(0.231)	(0.247)	(0.245)
				
Sex	−0.414	−0.131	−0.422	−0.144
	(0.565)	(0.595)	(0.559)	(0.599)
				
Body Mass Index	0.434 *	0.513 *	0.269	0.374
	(0.220)	(0.233)	(0.233)	(0.232)
				
SRsmoke	0.014	−0.441	0.037	−0.299
	(0.292)	(0.344)	(0.283)	(0.343)
				
HAC/ATS		1.111 ***		1.020 ***
		(0.260)		(0.270)
				
NIllnesses			3.058 *	2.883 *
			(1.457)	(1.461)
				
Constant	−3.449 ***	−3.766 ***	−5.798 ***	−5.939 ***
	(0.436)	(0.488)	(1.446)	(1.458)
Pseudo-R^2^	4.449	4.766	6.798	6.939

* *p* < 0.05, ** *p* < 0.01, *** *p* < 0.001. Note: SRsmoke = Self-reported smoking; HAC/ATS = Alcohol T-score; NIllnesses = Number of Chronic Illnesses.

**Table 5:** Firth regression showing that HAC (ATS) is a significant predictor of mortality in the ProSAAF sample, accounting for significant variance beyond control variables and beyond the presence of six specific Chronic Illnesses: cancer, liver disease, heart disease, diabetes, high blood pressure, and kidney disease; with cancer, blood pressure, and kidney disease contributing additional unique variance in mortality. cancer, liver disease, heart disease, diabetes, high blood pressure, or kidney disease (N = 336).

	Baseline	Model 1	Model 2	Model 3
	*β* (SE)	*β* (SE)	*β* (SE)	*β* (SE)
Age	0.595 **	0.440	0.391	0.292
	(0.227)	(0.231)	(0.264)	(0.274)
				
Sex	0.414	0.131	0.268	0.022
	(0.565)	(0.595)	(0.600)	(0.660)
				
Body Mass Index	0.434 *	0.513 *	0.267	0.362
	(0.220)	(0.233)	(0.253)	(0.260)
				
SRsmoke	0.014	−0.441	0.126	−0.211
	(0.292)	(0.344)	(0.298)	(0.335)
				
HAC/ATS		1.111 ***		1.019 ***
		(0.260)		(0.276)
				
Cancer			3.815 *	4.418 **
			(1.678)	(1.667)
				
Liver			4.426 *	3.877
			(2.246)	(2.272)
				
Heart Disease			0.154	0.326
			(0.762)	(0.863)
				
Diabetes			−0.435	−0.596
			(0.650)	(0.713)
				
High Blood Pressure			3.643 *	3.532 *
			(1.703)	(1.692)
				
Kidney			1.760 *	1.806 *
			(0.740)	(0.818)
				
Constant	−3.449 ***	−3.766 ***	−6.312 ***	−6.516 ***
	(0.436)	(0.488)	(1.689)	(1.655)
Pseudo-R^2^	4.449	4.766	7.312	7.516

* *p* < 0.05, ** *p* < 0.01, *** *p* < 0.001. Note: SRsmoke = Self-reported smoking; HAC/ATS = Alcohol T-score.

## Data Availability

Although the data are not publicly available due to potential problems with identifiability, the analytic code is available in Appendix B.

## Abbreviations

The following abbreviations are used in this manuscript:

HAC	Heavy Alcohol Consumption
FACHS	Family and Community Health Study
ProSAAF	Promoting Strong African American Families
ATS	Alcohol T-score
PCs	primary caregivers
BMI	Body Mass Index
MSdPCR	methylation-sensitive digital PCR
MLE	maximum likelihood estimation
SRHAC	Self-reported heavy alcohol consumption/non-self-report measure
HAC/ATS	Heavy Alcohol Consumption/Alcohol T-score
SRsmoke	Self-reported smoking
NIllnesses	Number of Chronic Illnesses
SD	Standard Deviation
AUD	alcohol use disorder
HBP	High Blood Pressure

## Appendix A

**Table A1:** Correlation matrix for study variables for the FACHS Sample with N = 360 including individual Chronic Illnesses.

	**1**	**2**	**3**	**4**	**5**	**6**	**7**	**8**	**9**	**10**	**11**	**12**	13	14
1. Age_40	—													
2. Sex	0.004	—												
3. BMI	−0.067	0.004	—											
4. HAC/ATS	0.174 **	−0.013	−0.035	—										
5. SRsmoke	−0.012	0.089 ^†^	−0.078	0.268 **	—									
6. NIllnesses	0.355 **	0.005	0.039	0.107 *	−0.036	—								
7. Mortality	0.470 **	−0.034	−0.130 *	0.260 **	0.038	0.236 **	—							
8. HBP	0.349 **	0.043	0.102 ^†^	0.034	−0.003	0.245 **	0.233 **	—						
9. Cancer	0.123 *	−0.045	−0.019	0.127 *	0.007	0.368 **	0.212 **	0.109 *	—					
10. Liver	0.100 ^†^	−0.027	0.095 ^†^	−0.029	−0.002	0.222 **	0.019	0.088^+^	0.077	—				
11. Heart Disease	0.219 **	0.019	0.058	0.146 **	−0.037	0.586 **	0.152 **	0.183 **	0.189 **	0.238 **	—			
12. Diabetes	0.400 **	−0.010	0.058	0.041	−0.017	0.717 **	0.229 **	0.236 **	0.098 ^†^	0.067	0.134 *	—		
13. Kidney	0.070	0.034	−0.056	0.039	−0.049	0.330 **	0.066	0.135 *	0.175 **	0.091 ^†^	0.224 **	0.127 *	—	
14. SRHAC	−0.030	0.093^†^	−0.082	−0.005	0.120 *	−0.003	−0.053	−0.110 *	−0.048	−0.029	−0.046	−0.067	0.156 **	—
Mean	47.125	0.042	35.018	−0.020	2.431	0.256	0.119	0.497	0.044	0.017	0.106	0.150	0.036	0.067
SD	8.119	0.200	8.733	0.047	5.705	0.437	0.325	0.501	0.206	0.128	0.308	0.358	0.187	0.300

^†^ *p* < 0.1, * *p* < 0.05, ** *p* < 0.01. Note: BMI = Body Mass Index; HAC/ATS = Alcohol T-score; SRsmoke = Self-reported smoking; NIllnesses = Number of Chronic Illnesses; HBP = high blood pressure; SRHAC = Self-reported heavy alcohol consumption; SD = Standard Deviation.

**Table A2:** Correlation matrix for study variables for the ProSAAF Sample with N = 336 including individual Chronic Illnesses.

	**1**	**2**	**3**	**4**	**5**	**6**	**7**	**8**	**9**	**10**	**11**	**12**	13	14
1. Age_40	—													
2. Sex	−0.211 **	—												
3. BMI	−0.091 ^†^	0.201 **	—											
4. HAC/ATS	0.139 *	−0.214 **	−0.174 **	—										
5. SRsmoke	−0.084	−0.198 **	−0.191 **	0.442 **	—									
6. NIllnesses	0.276 **	0.109 *	0.115 *	0.110 *	−0.009	—								
7. Mortality	0.145 **	−0.050	0.074	0.238 **	−0.030	0.136 *	—							
8. HBP	0.321 **	0.021	0.202 **	0.027	−0.083	0.338 **	0.229 **	—						
9. Cancer	0.089	−0.066	−0.086	0.045	0.140 *	0.203 **	0.114 *	−0.045	—					
1. Liver	0.063	0.070	−0.035	0.077	0.052	0.143 **	−0.016	−0.070	−0.008	—				
11. Heart Disease	0.238 **	−0.122 *	0.181 **	0.027	0.040	0.490 **	0.125 *	0.267 **	−0.029	−0.020	—			
12. Diabetes	0.159 **	−0.091 ^†^	0.081	0.103 ^†^	−0.045	0.827 **	0.067	0.283 **	0.025	−0.035	0.204 **	—		
13. Kidney	0.085	−0.052	0.019	0.055	−0.054	0.324 **	0.226 **	0.193 **	−0.019	−0.014	0.166 **	0.157 **	—	
14. SRHAC	−0.081	0.135 *	−0.075	0.209 **	0.342 **	−0.032	−0.040	−0.003	0.041	−0.042	−0.055	−0.027	−0.052	—
Mean	44.644	0.453	34.690	2.195	0.679	0.226	0.042	0.452	0.012	0.006	0.065	0.167	0.030	0.443
SD	8.265	0.498	9.072	3.527	1.235	0.419	0.200	0.498	0.109	0.077	0.248	0.373	0.170	0.827

^†^ *p* < 0.1, * *p* < 0.05, ** *p* < 0.01. Note: BMI = Body Mass Index; HAC/ATS = Alcohol T-score; SRsmoke = Self-reported smoking; NIllnesses = Number of Chronic Illnesses; HBP = high blood pressure; SRHAC = Self-reported heavy alcohol consumption; SD = Standard Deviation.

**Table A3:** Correlation matrix for study variables for the ProSAAF Sample with N = 336 including treatment condition to show that treatment condition was not significantly associated with other study variables.

	**1**	**2**	**3**	**4**	**5**	**6**	**7**	**8**	**9**
1. Age_40	—								
2. Sex	0.211 **	—							
3. BMI	−0.091 ^†^	0.201 **	—						
4. HAC/ATS	0.139 *	−0.214 **	−0.174 **	—					
5. SRsmoke	−0.084	−0.198 **	−0.191 **	0.442 **	—				
6. NIllnesses	0.276 **	0.109 *	0.115 *	0.110 *	−0.009	—			
7. Mortality	0.145 **	−0.050	0.074	0.238 **	−0.030	0.136 *	—		
8. SRHAC	−0.081	−0.135 *	−0.075	0.209 **	0.342 **	−0.032	−0.040	—	
9. Treatment Condition	0.024	−0.024	−0.031	−0.016	−0.054	−0.029	−0.063	−0.056	—
Mean	44.643	0.453	34.690	2.195	0.679	0.226	0.042	0.443	0.435
SD	8.265	0.498	9.072	3.527	1.235	0.419	0.200	0.827	0.496

^†^ *p* < 0.1, * *p* < 0.05, ** *p* < 0.01. Note: BMI = Body Mass Index; HAC/ATS = Alcohol T-score; SRsmoke = Self-reported smoking; NIllnesses = Number of Chronic Illnesses; SRHAC = Self-reported heavy alcohol consumption; SD = Standard Deviation.

**Table A4:** HAC/ATS is a more potent predictor of mortality than BMI, smoking, Self-reported HAC or Number of Chronic Illnesses in the FACHS sample, Self-reported HAC is not a significant predictor (N = 360).

	Baseline	Model 1	Model 2	Model 3
	*β* (SE)	*β* (SE)	*β* (SE)	*β* (SE)
Age	1.151 **	1.080 **	1.016 **	0.928 **
	(0.178)	(0.184)	(0.186)	(0.191)
Sex	−0.006	0.359	−0.072	0.317
	(0.913)	(0.886)	(0.927)	(0.890)
Body Mass Index	−0.425 *	−0.373	−0.472 *	−0.422
	(0.217)	(0.215)	(0.225)	(0.223)
SRsmoke	0.204	−0.016	0.209	−0.005
	(0.158)	(0.190)	(0.153)	(0.183)
SRHAC	−0.197	−0.088	−0.140	0.028
	(0.636)	(0.596)	(0.646)	(0.596)
HAC/ATS		0.715 **		0.743 **
		(0.219)		(0.217)
NIllnesses			0.857	1.019 *
			(0.486)	(0.504)
Constant	−2.432 **	−2.576 **	−2.979 **	−3.247 **
	(0.229)	(0.248)	(0.422)	(0.457)
Pseudo-R^2^	3.432	3.576	3.979	4.247

* *p* < 0.05, ** *p* < 0.01. Note: SRsmoke = Self-reported smoking; SRHAC = Self-reported heavy alcohol consumption; HAC/ATS = Alcohol T-score; NIllnesses = Number of Chronic Illnesses.

**Table A5:** HAC/ATS is a more potent predictor of mortality than BMI, smoking, self-reported HAC, or Number of Chronic Illnesses in the ProSAAF sample (N = 336).

	Baseline	Model 1	Model 2	Model 3
	*β* (SE)	*β* (SE)	*β* (SE)	*β* (SE)
Age	0.578 *	0.430	0.351	0.282
	(0.227)	(0.230)	(0.249)	(0.245)
				
Sex	0.436	0.154	0.438	0.149
	(0.566)	(0.597)	(0.561)	(0.599)
				
Body Mass Index	0.429	0.500 *	0.261	0.366
	(0.221)	(0.234)	(0.232)	(0.232)
				
SRsmoke	0.057	−0.350	0.059	−0.261
	(0.306)	(0.356)	(0.292)	(0.349)
				
SRHAC	−0.092	−0.185	−0.048	−0.058
	(0.441)	(0.452)	(0.395)	(0.366)
				
HAC/ATS		1.112 **		1.019 **
		(0.260)		(0.271)
				
NIllnesses			3.067 *	2.879 *
			(1.457)	(1.459)
				
Constant	−3.381 ***	−3.658 ***	−5.752 ***	−5.871 ***
	(0.457)	(0.503)	(1.452)	(1.465)
Pseudo-R^2^	4.381	4.658	6.752	6.871

* *p* < 0.05, ** *p* < 0.01, *** *p* < 0.001. Note: SRsmoke = Self-reported smoking; SRHAC = Self-reported heavy alcohol consumption; HAC/ATS = Alcohol T-score; NIllnesses = Number of Chronic Illnesses.

**Table A6:** HAC/ATS is a stronger predictor of mortality over a three year period than presence of six specific Chronic Illnesses in the ProSAAF sample: cancer, liver disease, heart disease, diabetes, high blood pressure, or kidney disease. Self-reported HAC (SRHAC) does not contribute to prediction (N = 336).

	Baseline	Model 1	Model 2	Model 3
	*β* (SE)	*β* (SE)	*β* (SE)	*β* (SE)
Age	0.578 *	0.430	0.369	0.272
	(0.227)	(0.230)	(0.266)	(0.273)
Sex	0.436	0.154	0.292	0.044
	(0.566)	(0.597)	(0.605)	(0.662)
Body Mass Index	0.429	0.500 *	0.261	0.356
	(0.221)	(0.234)	(0.253)	(0.259)
SRsmoke	0.057	−0.350	0.144	−0.179
	(0.306)	(0.356)	(0.314)	(0.346)
SRHAC	−0.092	−0.185	−0.015	−0.048
	(0.441)	(0.452)	(0.414)	(0.404)
HAC/ATS		1.112 ***		1.016 ***
		(0.260)		(0.277)
Cancer			3.743 *	4.356 **
			(1.683)	(1.659)
Liver			4.390	3.793
			(2.254)	(2.269)
Heart Disease			0.133	0.293
			(0.759)	(0.861)
Diabetes			−0.416	−0.577
			(0.650)	(0.718)
High Blood Pressure			3.652 *	3.501 *
			(1.694)	(1.666)
Kidney			1.719 *	1.760 *
			(0.738)	(0.815)
Constant	−3.381 ***	−3.658 ***	−6.274 ***	−6.420 ***
	(0.457)	(0.503)	(1.688)	(1.649)
Pseudo-R^2^	4.381	4.658	7.274	7.420

* *p* < 0.05, ** *p* < 0.01, *** *p* < 0.001. Note: SRsmoke = Self-reported smoking; SRHAC = Self-reported heavy alcohol consumption; HAC/ATS = Alcohol T-score.

**Table A7:** HAC/ATS is a stronger predictor of mortality over a three-year period than presence of six specific Chronic Illnesses in the ProSAAF sample: cancer, liver disease, heart disease, diabetes, high blood pressure, or kidney disease (N = 336). Inclusion of ProSAAF relationship enhancement conditions does not affect prediction of mortality, nor does inclusion of self-reported HAC.

	Baseline	Model 1	Model 2	Model 3
	*β* (SE)	*β* (SE)	*β* (SE)	*β* (SE)
Age	0.556 *	0.406	0.301	0.117
	(0.223)	(0.229)	(0.269)	(0.290)
Sex	0.447	0.080	0.291	0.015
	(0.564)	(0.602)	(0.603)	(0.671)
Body Mass Index	0.403	0.467 *	0.241	0.307
	(0.220)	(0.237)	(0.240)	(0.270)
SRsmoke	0.027	−0.386	0.117	−0.216
	(0.304)	(0.357)	(0.311)	(0.359)
Treatment Condition	−0.606	−0.573	−1.223	−1.509
	(0.582)	(0.626)	(0.690)	(0.810)
SRHAC	−0.121	−0.189	−0.059	−0.051
	(0.454)	(0.460)	(0.443)	(0.391)
HAC/ATS		1.109 ***		1.056 ***
		(0.265)		(0.297)
Cancer			3.566 *	4.049 *
			(1.746)	(1.670)
Liver			4.236	4.385
			(2.261)	(2.295)
Heart Disease			0.158	0.315
			(0.770)	(0.901)
Diabetes			−0.644	−0.946
			(0.677)	(0.753)
High Blood Pressure			3.652 *	3.854 *
			(1.597)	(1.696)
Kidney			2.296 **	2.487 **
			(0.843)	(0.927)
Constant	−3.109 **	−3.351 ***	−5.727 ***	−6.047 ***
	(0.493)	(0.552)	(1.591)	(1.664)
Pseudo-R^2^	4.109	4.351	6.727	7.047

* *p* < 0.05, ** *p* < 0.01, *** *p* < 0.001. Note: SRsmoke = Self-reported smoking; SRHAC = Self-reported heavy alcohol consumption; HAC/ATS = Alcohol T-score.

## Appendix B

Analytic Code Used in data analyses

****************************Table 1****************************

*******************************************************

* FACHS (Below) + ProSAAF (Above) correlation table

*******************************************************

⁡

clear all

set more off

⁡

* Define these ONCE so they exist for both datasets

local myvars Age_40 SEX BMIW5 ATSW5 smokeW5 binge5 Mortality cancerW5 liverW5 HeartW5 DiabetesW5 HBPW5 KidneyW5

local vars Age_40 SEX BMIW5 ATSW5 smokeW5 binge5 illness_count Mortality

⁡

*******************************************************

* 0) Helper program to post b/V/p into e()-returns

*******************************************************

capture program drop postcorr

program define postcorr, eclass

version 13

syntax, b(name) v(name) p(name)

ereturn clear

ereturn post ‘b’ ‘v’

ereturn matrix p = ‘p’

end

⁡

⁡

*******************************************************

* 1) PROCESS FACHS DATA

*******************************************************

use “C:\Mortality.dta”, clear

⁡

replace Mortality =. if Mortality == 99

⁡

local myvars Age_40 SEX ATSW5 BMIW5 smokeW5 binge5 Mortality cancerW5 liverW5 HeartW5 DiabetesW5 HBPW5 KidneyW5

local vars Age_40 SEX BMIW5 ATSW5 smokeW5 binge5 illness_count Mortality

⁡

capture drop illness_count

gen illness_count = cancerW5 + liverW5 + HeartW5 + DiabetesW5 + KidneyW5

⁡

* Convert to binary: 1 if they have any conditions, 0 otherwise

replace illness_count = (illness_count > 0) if !missing(illness_count)

⁡

pwcorr ‘vars’, sig

matrix Rho_F = r(C)

matrix P_F = r(sig)

⁡

sum Age_40 SEX BMIW5 ATSW5 smokeW5 binge5 illness_count Mortality

⁡

*******************************************************

* 2) PROCESS ProSAAF DATA

*******************************************************

set more off

use “C:\Mortality-ATS.dta”, clear

⁡

replace Mortality = . if Mortality == 99

recode SEX (1=1) (2=0)

⁡

local myvars Age_40 SEX ATSW5 BMIW5 smokeW5 binge5 Mortality cancerW5 liverW5 HeartW5 DiabetesW5 HBPW5 KidneyW5

local vars Age_40 SEX BMIW5 ATSW5 smokeW5 binge5 illness_count Mortality

⁡

⁡

capture drop illness_count

gen illness_count = cancerW5 + liverW5 + HeartW5 + DiabetesW5 + KidneyW5

⁡

* Convert to binary: 1 if they have any conditions, 0 otherwise

replace illness_count = (illness_count > 0) if !missing(illness_count)

⁡

pwcorr ‘vars’, sig

matrix Rho_P = r(C)

matrix P_P = r(sig)

⁡

*******************************************************

* 3) COMBINE MATRICES: FACHS lower/ProSAAF upper

*******************************************************

local k: word count ‘vars’

⁡

matrix Combined_R = J(‘k’, ‘k’, .)

matrix Combined_P = J(‘k’, ‘k’, .)

⁡

forval r = 1/‘k’ {

forval c = 1/‘k’ {

if ‘r’ == ‘c’ {

matrix Combined_R[‘r’,‘c’] = 1

matrix Combined_P[‘r’,‘c’] = .

}

else if ‘r’ > ‘c’ {

matrix Combined_R[‘r’,‘c’] = Rho_F[‘r’,‘c’]

matrix Combined_P[‘r’,‘c’] = P_F[‘r’,‘c’]

}

else {

matrix Combined_R[‘r’,‘c’] = Rho_P[‘r’,‘c’]

matrix Combined_P[‘r’,‘c’] = P_P[‘r’,‘c’]

}

}

}

⁡

⁡

⁡

*******************************************************

* 4) CLEAN VARIABLE LABELS

*******************************************************

label variable Age_40 “Age”

label variable SEX “Sex”

label variable BMIW5 “BMI”

label variable ATSW5 “HAC/ATS”

label variable smokeW5 “SRsmoke”

label variable illness_count “Number of Chronic Illnesses”

label variable Mortality “Mortality”

label variable binge5 “SRHAC”

⁡

*******************************************************

* 5) RESHAPE MATRICES INTO e(b) FORMAT FOR esttab

*******************************************************

tempname b_vec p_vec

matrix ‘b_vec’ = vec(Combined_R)’

matrix ‘p_vec’ = vec(Combined_P)’

⁡

local coefnames ““

local eqnames ““

⁡

forval r = 1/‘k’ {

local rowvar: word ‘r’ of ‘vars’

forval c = 1/‘k’ {

local colvar: word ‘c’ of ‘vars’

local coefnames “‘coefnames’ ‘colvar’“

local eqnames “‘eqnames’ ‘rowvar’“

}

}

⁡

matrix colnames ‘b_vec’ = ‘coefnames’

matrix coleq ‘b_vec’ = ‘eqnames’

⁡

matrix colnames ‘p_vec’ = ‘coefnames’

matrix coleq ‘p_vec’ = ‘eqnames’

⁡

local total_cells = colsof(‘b_vec’)

tempname VV

matrix ‘VV’ = J(‘total_cells’, ‘total_cells’, 0)

matrix colnames ‘VV’ = ‘coefnames’

matrix coleq ‘VV’ = ‘eqnames’

matrix rownames ‘VV’ = ‘coefnames’

matrix roweq ‘VV’ = ‘eqnames’

⁡

*******************************************************

* 6) POST + EXPORT

*******************************************************

eststo clear

postcorr, b(‘b_vec’) v(‘VV’) p(‘p_vec’)

⁡

esttab . using “correlation_Table1.rtf”, replace///

cells(“b(fmt(3) star)”)///

starlevels(+ 0.10 * 0.05 ** 0.01)///

unstack///

noobs nonumber label///

title(“Table 1: FACHS (Below) and ProSAAF (Above) Correlations”)///

addnotes(“Note: FACHS (Lower), ProSAAF (Upper). + *p* < 0.10, * *p* < 0.05, ** *p* < 0.01”)

⁡

****************************Table 2****************************

*******************************************************

* Firth Model: ATSW5 predicted Mortality (FACHS)

*******************************************************

⁡

set more off

use “C:\Mortality.dta”,clear

⁡

replace Mortality = . if Mortality == 99

⁡

* Define the variable list for convenience

local myvars Age_40 SEX ATSW5 BMIW5 smokeW5 cancerW5 liverW5 HeartW5 DiabetesW5 HBPW5 KidneyW5

⁡

* 1. Check how many missing values exist for each variable

misstable summarize ‘myvars’

⁡

* 2. See the pattern of missingness (to see if people miss multiple questions)

misstable patterns ‘myvars’

⁡

* 3. Visual check (requires the ‘mdesc’ package: ssc install mdesc)

mdesc Age_40 SEX ATSW5 BMIW5 smokeW5 cancerW5 liverW5 HeartW5 DiabetesW5 HBPW5 KidneyW5

⁡

* Keep only observations with no missing data in these specific variables

egen missing_count = rowmiss(Age_40 SEX ATSW5 BMIW5 smokeW5 cancerW5 liverW5 HeartW5 DiabetesW5 HBPW5 KidneyW5)

keep if missing_count == 0

⁡

* Standardize continuous variables (z-scores)

foreach v in Age_40 BMIW5 ATSW5 smokeW5 {

egen z‘v’ = std(‘v’)

}

⁡

* Sum the conditions

egen chronic_health = rowtotal(cancerW5 liverW5 HeartW5 DiabetesW5 HBPW5 KidneyW5)

⁡

* Convert to binary: 1 if they have any conditions, 0 otherwise

replace chronic_health = (chronic_health > 0) if !missing(chronic_health)

⁡

* Add labels for clarity

label variable chronic_health “Chronic Illness”

⁡

⁡

* Define a program to run firthlogit and add McFadden pseudo-R2

capture program drop firth_r2

program define firth_r2, eclass

version 18

syntax varlist(min = 2 numeric fv) [if] [in] [, *]

⁡

* first var is depvar, rest are predictors

gettoken y x: varlist

⁡

* Full model

quietly firthlogit ‘y’ ‘x’ ‘if’ ‘in’, ‘options’

local ll_full = e(ll)

⁡

* Null model on the SAME estimation sample

tempvar esamp

gen byte ‘esamp’ = e(sample)

quietly firthlogit ‘y’ if ‘esamp’, ‘options’

local ll_null = e(ll)

⁡

* Refit full model so e(b) and e(V) correspond to the model you want to tabulate

quietly firthlogit ‘y’ ‘x’ if ‘esamp’, ‘options’

⁡

* Add pseudo-R2 as an e()-scalar so esttab/estadd can see it

ereturn scalar pr2 = 1−(‘ll_full’/‘ll_null’)

end

⁡

*Baseline model

firthlogit Mortality zAge_40 SEX zBMIW5 zsmokeW5

estadd scalar pr2 = 1−(‘ll_full’/‘ll_null’)

est store M0

⁡

*Model 1

firthlogit Mortality zAge_40 SEX zBMIW5 zsmokeW5 zATSW5

estadd scalar pr2 = 1−(‘ll_full’/‘ll_null’)

est store M1

⁡

*Model 2

firthlogit Mortality zAge_40 SEX zBMIW5 zsmokeW5 chronic_health

estadd scalar pr2 = 1−(‘ll_full’/‘ll_null’)

est store M2

⁡

*Model 3

firthlogit Mortality zAge_40 SEX zBMIW5 zsmokeW5 chronic_health zATSW5

estadd scalar pr2 = 1−(‘ll_full’/‘ll_null’)

est store M3

⁡

esttab M0 M1 M2 M3///

using Table2_FACHS.rtf,///

replace///

b(%9.3f) se(%9.3f)///

star(* 0.05 ** 0.01 *** 0.001)///

stats(pr2, labels(“R-squared” fmt(0 3)))///

label///

mtitles(“Baseline” “Model 1” “Model 2” “Model 3”)

⁡

⁡

****************************Table 3****************************

*******************************************************

* the chronic health conditions as separate predictors

*******************************************************

set more off

⁡

use “C:\Mortality.dta”, clear

⁡

⁡

replace Mortality = . if Mortality == 99

⁡

⁡

* Define the variable list for convenience

local myvars Age_40 SEX ATSW5 BMIW5 smokeW5 cancerW5 liverW5 HeartW5 DiabetesW5 HBPW5 KidneyW5

⁡

* 1. Check how many missing values exist for each variable

misstable summarize ‘myvars’

⁡

* 2. See the pattern of missingness (to see if people miss multiple questions)

misstable patterns ‘myvars’

⁡

* ssc install mdesc

⁡

* 3. Visual check (requires the ‘mdesc’ package: ssc install mdesc)

mdesc Age_40 SEX ATSW5 BMIW5 smokeW5 cancerW5 liverW5 HeartW5 DiabetesW5 HBPW5 KidneyW5

⁡

* Keep only observations with no missing data in these specific variables

egen missing_count = rowmiss(Age_40 SEX ATSW5 BMIW5 smokeW5 cancerW5 liverW5 HeartW5 DiabetesW5 HBPW5 KidneyW5)

keep if missing_count == 0

⁡

* Standardize continuous variables (z-scores)

foreach v in Age_40 BMIW5 ATSW5 smokeW5 {

egen z‘v’ = std(‘v’)

}

⁡

* Define a program to run firthlogit and add McFadden pseudo-R2

capture program drop firth_r2

program define firth_r2, eclass

version 18

syntax varlist(min = 2 numeric fv) [if] [in] [, *]

⁡

* first var is depvar, rest are predictors

gettoken y x: varlist

⁡

* Full model

quietly firthlogit ‘y’ ‘x’ ‘if’ ‘in’, ‘options’

local ll_full = e(ll)

⁡

* Null model on the SAME estimation sample

tempvar esamp

gen byte ‘esamp’ = e(sample)

quietly firthlogit ‘y’ if ‘esamp’, ‘options’

local ll_null = e(ll)

⁡

* Refit full model so e(b) and e(V) correspond to the model you want to tabulate

quietly firthlogit ‘y’ ‘x’ if ‘esamp’, ‘options’

⁡

* Add pseudo-R2 as an e()-scalar so esttab/estadd can see it

ereturn scalar pr2 = 1-(‘ll_full’/‘ll_null’)

end

⁡

⁡

*Baseline model

firthlogit Mortality zAge_40 SEX zBMIW5 zsmokeW5

estadd scalar pr2 = 1-(‘ll_full’/‘ll_null’)

est store TM0

⁡

*Model 1

firthlogit Mortality zAge_40 SEX zBMIW5 zsmokeW5 zATSW5

estadd scalar pr2 = 1-(‘ll_full’/‘ll_null’)

est store TM1

⁡

*Model 2

firthlogit Mortality zAge_40 SEX zBMIW5 zsmokeW5 cancerW5 liverW5 HeartW5 DiabetesW5 HBPW5 KidneyW5

estadd scalar pr2 = 1-(‘ll_full’/‘ll_null’)

est store TM2

⁡

*Model 3

firthlogit Mortality zAge_40 SEX zBMIW5 zsmokeW5 zATSW5 cancerW5 liverW5 HeartW5 DiabetesW5 HBPW5 KidneyW5

estadd scalar pr2 = 1-(‘ll_full’/‘ll_null’)

est store TM3

⁡

esttab TM0 TM1 TM2 TM3///

using Table3.rtf,///

replace///

b(%9.3f) se(%9.3f)///

star(* 0.05 ** 0.01 *** 0.001)///

stats(pr2, labels(“Pseudo-R-squared” fmt(0 3)))///

label///

mtitles(“Baseline” “Model 1” “Model 2” “Model 3”)

****************************Table 4****************************

*******************************************************

* Firth Model: ATSW5 predicted Mortality (ProSAAF)

*******************************************************

⁡

set more off

use “C:\Mortality-ATS.dta”,clear

⁡

replace Mortality = . if Mortality == 99

recode SEX (1=1) (2=0)

⁡

* Define the variable list for convenience

local myvars Age_40 SEX ATSW5 BMIW5 smokeW5 cancerW5 liverW5 HeartW5 DiabetesW5 HBPW5 KidneyW5

⁡

* 1. Check how many missing values exist for each variable

misstable summarize ‘myvars’

⁡

* 2. See the pattern of missingness (to see if people miss multiple questions)

misstable patterns ‘myvars’

⁡

* 3. Visual check (requires the ‘mdesc’ package: ssc install mdesc)

mdesc Age_40 SEX ATSW5 BMIW5 smokeW5 cancerW5 liverW5 HeartW5 DiabetesW5 HBPW5 KidneyW5

⁡

* Keep only observations with no missing data in these specific variables

egen missing_count = rowmiss(Age_40 SEX ATSW5 BMIW5 smokeW5 cancerW5 liverW5 HeartW5 DiabetesW5 HBPW5 KidneyW5 Mortality)

keep if missing_count == 0

⁡

sum Age_40 SEX ATSW5 BMIW5 smokeW5 cancerW5 liverW5 HeartW5 DiabetesW5 HBPW5 KidneyW5 Mortality

⁡

⁡

* Standardize continuous variables (z-scores)

foreach v in Age_40 BMIW5 ATSW5 smokeW5 {

egen z‘v’ = std(‘v’)

}

⁡

* Sum the conditions

egen chronic_health = rowtotal(cancerW5 liverW5 HeartW5 DiabetesW5 HBPW5 KidneyW5)

⁡

* Convert to binary: 1 if they have any conditions, 0 otherwise

replace chronic_health = (chronic_health > 0) if !missing(chronic_health)

⁡

* Add labels for clarity

label variable chronic_health “Chronic Illness”

⁡

* Define a program to run firthlogit and add McFadden pseudo-R2

capture program drop firth_r2

program define firth_r2, eclass

version 18

syntax varlist(min = 2 numeric fv) [if] [in] [, *]

⁡

* first var is depvar, rest are predictors

gettoken y x: varlist

⁡

* Full model

quietly firthlogit ‘y’ ‘x’ ‘if’ ‘in’, ‘options’

local ll_full = e(ll)

⁡

* Null model on the SAME estimation sample

tempvar esamp

gen byte ‘esamp’ = e(sample)

quietly firthlogit ‘y’ if ‘esamp’, ‘options’

local ll_null = e(ll)

⁡

* Refit full model so e(b) and e(V) correspond to the model you want to tabulate

quietly firthlogit ‘y’ ‘x’ if ‘esamp’, ‘options’

⁡

* Add pseudo-R2 as an e()-scalar so esttab/estadd can see it

ereturn scalar pr2 = 1-(‘ll_full’/‘ll_null’)

end

⁡

* Baseline model

firthlogit Mortality zAge_40 SEX zBMIW5 zsmokeW5

estadd scalar pr2 = 1-(‘ll_full’/‘ll_null’)

est store M0

⁡

* Model 1

firthlogit Mortality zAge_40 SEX zBMIW5 zsmokeW5 zATSW5

estadd scalar pr2 = 1-(‘ll_full’/‘ll_null’)

est store M1

⁡

* Model 2

firthlogit Mortality zAge_40 SEX zBMIW5 zsmokeW5 chronic_health

estadd scalar pr2 = 1-(‘ll_full’/‘ll_null’)

est store M2

⁡

* Model 3

firthlogit Mortality zAge_40 SEX zBMIW5 zsmokeW5 zATSW5 chronic_health

estadd scalar pr2 = 1-(‘ll_full’/‘ll_null’)

est store M3

⁡

esttab M0 M1 M2 M3///

using Table4.rtf,///

replace///

b(%9.3f) se(%9.3f)///

star(* 0.05 ** 0.01 *** 0.001)///

stats(pr2, labels(“Pseudo-R-squared” fmt(0 3)))///

label///

mtitles(“Baseline” “Model 1” “Model 2” “Model 3”)

****************************Table 5****************************

*******************************************************

* the chronic health conditions as separate predictors

*******************************************************

⁡

set more off

⁡

use “C:\Mortality-ATS.dta”, clear

⁡

replace Mortality = . if Mortality == 99

⁡

recode SEX (1=1) (2=0)

⁡

* Define the variable list for convenience

local myvars Age_40 SEX ATSW5 BMIW5 smokeW5 cancerW5 liverW5 HeartW5 DiabetesW5 HBPW5 KidneyW5

⁡

* 1. Check how many missing values exist for each variable

misstable summarize ‘myvars’

⁡

* 2. See the pattern of missingness (to see if people miss multiple questions)

misstable patterns ‘myvars’

⁡

* ssc install mdesc

⁡

* 3. Visual check (requires the ‘mdesc’ package: ssc install mdesc)

mdesc Age_40 SEX ATSW5 BMIW5 smokeW5 cancerW5 liverW5 HeartW5 DiabetesW5 HBPW5 KidneyW5

⁡

⁡

* Standardize continuous variables (z-scores)

foreach v in Age_40 BMIW5 ATSW5 smokeW5 {

egen z‘v’ = std(‘v’)

}

⁡

* Define a program to run firthlogit and add McFadden pseudo-R2 (same sample)

capture program drop firth_r2

program define firth_r2, eclass

version 18

syntax varlist(min = 2 numeric fv) [if] [in] [, *]

⁡

* first var is depvar, rest are predictors

gettoken y x: varlist

⁡

* Full model

quietly firthlogit ‘y’ ‘x’ ‘if’ ‘in’, ‘options’

local ll_full = e(ll)

⁡

* Null model on the SAME estimation sample

tempvar esamp

gen byte ‘esamp’ = e(sample)

quietly firthlogit ‘y’ if ‘esamp’, ‘options’

local ll_null = e(ll)

⁡

* Refit full model so e(b) and e(V) correspond to the model you want to tabulate

quietly firthlogit ‘y’ ‘x’ if ‘esamp’, ‘options’

⁡

* Add pseudo-R2 as an e()-scalar so esttab/estadd can see it

ereturn scalar pr2 = 1-(‘ll_full’/‘ll_null’)

end

⁡

*Baseline model

firthlogit Mortality zAge_40 SEX zBMIW5 zsmokeW5

estadd scalar pr2 = 1-(‘ll_full’/‘ll_null’)

est store TM0

⁡

*Model 1

firthlogit Mortality zAge_40 SEX zBMIW5 zsmokeW5 zATSW5

estadd scalar pr2 = 1-(‘ll_full’/‘ll_null’)

est store TM1

⁡

*Model 2

firthlogit Mortality zAge_40 SEX zBMIW5 zsmokeW5 cancerW5 liverW5 HeartW5 DiabetesW5 HBPW5 KidneyW5

estadd scalar pr2 = 1-(‘ll_full’/‘ll_null’)

est store TM2

⁡

*Model 3

firthlogit Mortality zAge_40 SEX zBMIW5 zsmokeW5 zATSW5 cancerW5 liverW5 HeartW5 DiabetesW5 HBPW5 KidneyW5

estadd scalar pr2 = 1-(‘ll_full’/‘ll_null’)

est store TM3

⁡

esttab TM0 TM1 TM2 TM3///

using Table5.rtf,///

replace///

b(%9.3f) se(%9.3f)///

star(* 0.05 ** 0.01 *** 0.001)///

stats(pr2, labels(“Pseudo-R-squared” fmt(0 3)))///

label///

mtitles(“Baseline” “Model 1” “Model 2” “Model 3”)

## Appendix C. Detailed Description of Sample and Procedure (FACHS)—See Also [14]

The Family and Community Health Study (FACHS) is a longitudinal study of 889 Black American families that began in 1997. FACHS used a stratified random-sampling design to recruit families from 1990 U.S. census block-group areas (BGAs) in Georgia and Iowa. BGAs were eligible if they met two criteria: (a) at least 10% Black American residents (the lowest density that allowed cost-effective door-to-door recruitment) and (b) meaningful variation in the proportion of families with children living below the federal poverty line. Eligible BGAs in both states formed a heterogeneous sampling frame that included rural communities, small towns, suburbs, and mid-sized cities. From this frame, FACHS randomly selected 259 BGAs—144 in Iowa and 115 in Georgia.

### Appendix C.1. Recruitment Procedures

Recruitment procedures followed protocols used in prior studies of Black American families. In Georgia, FACHS focused on 12 counties in northeast Georgia outside inner-city Atlanta; 10 of these counties had populations of 30,000 or fewer. To ensure representation of higher-SES contexts, additional BGAs were sampled from Athens and Atlanta suburbs. Within each selected BGA, a trusted community liaison compiled a roster of households with a Black American child aged 10–12, drawing on information from school personnel, clergy, youth groups, and local organizations. Families were then randomly selected and contacted via telephone or home visit; refusals were replaced until BGA quotas were met.

In Iowa, all BGAs meeting the ≥10% Black American threshold were located in Waterloo (population ≈ 65,000) and Des Moines (≈ 193,000). Public schools provided enrollment lists for Black American students in grades 4–6. Because only about 3% of students attended non-public schools in 1996/97 (Iowa Department of Education), coverage error was minimal. Households with an eligible child (fifth grade) were randomly sampled and invited by mail, followed by telephone recruitment.

### Appendix C.2. Participation and Baseline Sample

Across states, 61–68% of located, eligible families consented to participate—rates comparable to national probability samples of Black American adults and typical of studies involving low-income or minoritized populations. Families received a $100 honorarium per wave. The final baseline cohort included 889 families, with substantial variability in neighborhood racial composition and poverty. Post hoc comparisons indicated that the sample’s sociodemographic profile closely mirrored Black American families living in the targeted BGAs, supporting its representativeness for examining neighborhood influences on youth development. Youth completed the youth instrument directly, and caregivers completed the parent instrument; caregivers did not respond on behalf of youth. At baseline (1997), approximately half the sample resided in Georgia (N = 422) and half in Iowa (N = 467).

### Appendix C.3. Description of the Analytic Sample

We analyzed from Wave 5 of FACHS data 2007–2008, to characterize potential predictors of mortality in midlife. Because the identity of the primary caregiver could change across waves (e.g., the mother was identified as the primary caregiver at Wave 1; whereas, the grandmother was identified as the primary caregiver at Wave 2). Of the 889 primary caregivers recruited at baseline in 1997, 823 identified as African American (the remaining 66 identified with other racial groups). Within the African American primary-caregiver sample, 767 were women and 56 were men at baseline. In 2008, 642 African American primary caregivers (607 women and 35 men) were successfully re-interviewed and completed the psychosocial survey; blood collection began that year. Because of logistical and financial constraints, phlebotomy was offered only to participants who were still residing in Georgia or Iowa, and approximately two-thirds of eligible participants consented to a non-fasting blood draw.

Among the 607 African American women re-interviewed in 2008, we assayed whole blood to characterize genome-wide methylation for 469 women in 2008, and usable genome-wide methylation results were obtained for 379. For African American men, whole blood samples were available for 171 participants in 2008, and usable samples were obtained for 129.

Trained phlebotomists conducted morning home visits and collected 30 mL of blood (four tubes). Serum tubes were centrifuged on-site using portable centrifuges, and the separated sera were packed on dry ice and shipped to the Iowa Psychiatric Genetic Laboratory at the University of Iowa, where whole blood samples were plated and stored at −20 °C until analyzed.

All protocols and procedures were approved by the University Institutional Review Board (FACHS Weathering; Protocol #0000 4856).

### Appendix C.4. Detailed Description of the ProSAAF Sample—See Also [15]

ProSAAF is a longitudinal study of African American couples with a pre-adolescent or adolescent child that began in 2012. All participants lived in small towns and communities in Georgia in which poverty rates are among the highest in the nation and unemployment rates are above the national average. Of the 4552 families initially identified, 2655 were excluded due to non-response to the initial mailing (N = 768) and follow-up contacts attempts (e.g., phone calls) when such information was available (N = 1897). Thus, a total of 1897 families were screened for eligibility. Of these, 1551 families were excluded due to ineligibility (N = 1145), refusal (N = 347), and inability to schedule assessment (n = 59). This left 692 adults (from 346 families) at baseline assessment, and they were randomly assigned to the intervention (N = 344) or control (N = 348) condition.

Project staff visited participants’ homes, explained the study in greater detail, and obtained informed consent. Participants completed the baseline (Wave 1; W1) assessments using audio computer-assisted self-interview software installed on laptop computers. Families were visited for Waves 2, 3, and 4 (W2, W3, and W4) assessments a mean of 9.4 months, 17.0 months, and 24.5 months after baseline. For each follow-up assessment, project staff again visited participants’ homes, and participants completed measures on laptop computers. Each adult was compensated with a $50 check for completing each wave.

At the baseline interview, 63% were married, with an average marital duration of 9.7 years. Unmarried couples had been living together for 6.73 years. The mean age was 36.51 years for women and 39.98 years for men. The majority of the study participants could be classified as working poor: 51% had incomes below 100% of the federal poverty level, and an additional 17% had incomes between 100% and 150% of that level. Median monthly income was $1375 (SD = $1375; range $1 to $7500) for men and $1220 (SD = $1440; range $1 to $10,000) for women. Attrition, defined as instances in which no family member participated in the follow-up assessment, was 13.6% at W2, 12.1% at W3, and 12.7% at W4. Attrition was greater across waves for men than for women, but did not vary for primary indicators of family processes, or sociodemographic variables.

In 2018, 6 years after baseline assessment, we began recontacting participants regarding participation in a Wave 5 (W5) assessment to be focused on healthy aging of the adults in the sample (collected from 2018 to 2020 W5, all adults who had participated in prior waves were eligible to participate. Of those participating at W4 (i.e., 24-month follow-up n = 569), 70% (n = 395) agreed to participate at the six-year follow-up (W5). The mean age of participants at W5 was 44.61 years (women = 43.02 years; men = 46.49 years). The loss of data at Wave 5 relative to Wave 4 was due, in part, to the onset of the pandemic, which interrupted our Wave 5 assessment. As a result, after the onset of the pandemic, participants could not be visited at home for data collection as had been our practice up to that point, and so could not provide blood samples.

Of the adults analyzed in the current study, 63% percent were married, with an average marital duration of 9.7 years (range 0–56 years). Unmarried couples had been living together an average of 6.75 years (range 0.25–24). Men’s mean age was 39.73 (range 21–83) and women’s mean age was 36.54 (SD = 7.45; range 23–73). More than 99% adults were African American, including at least 1 member in every dyad. Men’s median education level was high school or GED (ranging from less than grade 9 to a doctorate or professional degree) and women’s median education level was some college or trade school (ranging from less than grade 9 to a master’s degree). The majority of men (74.1%; 65% full-time; 9% part) and women (61.0%; 45% full-time; 16% part) reported full- or part-time employment. Median monthly income was $1400 (range $1–$7500) for men and $1270 (range $1–$8900) for women). Total number of children residing in the home ranged from 1 to 8, with a mean of 2.99.

**ProSAAF implementation.** A trained African American facilitator visited each couple’s home for 6 consecutive weeks and facilitated a 2 h session with co-parenting adults and children. All facilitators had received 40 h of training in program content, facilitation and delivery methods, and adherence to the program manual. Facilitators were drawn from communities similar to those being served. All facilitators had either a bachelor degree or higher and/or had two or more years providing prevention programming or home visits in rural Georgia. Families participated in six primary sessions (with 97% participating in all six). The facilitator guided couples through video instruction and modeling, structured activities, and specific topics for discussion. The first 60 min of each session focused on the couple’s relationship. The next 30 min of each session focused on parenting topics (e.g., school, peers, children’s development, discipline). The facilitator then met with the target child for a youth activity (e.g., self-esteem, peer pressure, understanding parents) while the couple took a break in a different room. After the 15 min youth activity, the entire family came back together to meet with the facilitator for a 15 min family activity (e.g., discussions, games). A booster session was scheduled approximately 2 months after program completion and approximately 2 months before post-test assessment to reinforce material covered during the main course of instruction; 74.4% of intervention families participated. This implementation model included multiple components designed to achieve high rates of participation and retention among fathers and father figures, including refinement of engagement protocols and use of a home-based implementation model.

**Control group.** Couples in the control group were assessed on the same schedule as those in the intervention group, thereby controlling for effects of repeated measurement, maturation, individual differences, and external social changes. Couples were mailed the book, “12 Hours to A Great Marriage”, and an accompanying workbook after baseline. This book provides reasons for enhancing the marital relationship, guidelines and examples of communication and problem-solving strategies, and exercises that individuals and couples could implement to enhance their relationships.

**Wave 5 Biological Assessment**. Given our focus on methylation-based assessment of HAC, our analyzed sample includes only those who provided blood-based biomarker data at Wave 5 (six years after baseline). This resulted in a final sample of 348 at Wave 5. To collect blood samples at W5 a certified phlebotomist visited the home and collected six tubes of blood (30 mL) from each participant. We delivered blood samples to the Psychiatric Genetics Lab at the University of Iowa. DNA was prepared via standard procedures and stored at −20 °C until used. Epigenome-wide methylation assessments were conducted using Illumina InfiniumMethylationEpic Beadchip array (a.k.a. EPIC) by the University of Minnesota Genome Center according to the manufacturer’s (Illumina, San Diego) instructions. Samples were randomized on arrays to minimize batch effects. The resulting data were DASEN normalized using the *MethyLumi*, *WateRmelon* and *IlluminaHumanMethylationEPICanno. ilm10b2.hg19* (Illumina) R packages. CpG values were background-corrected using the “noob” method. Finally, data were subject to standard sample and probe level quality control measures. All laboratory analyses were performed by staff blinded to participants’ other study variables and couple identifiers to ensure analytical independence.
